# Favorable factors for the survival of ST-segment elevation myocardial infarction patients with medium- and high-risk thrombolysis in myocardial infarction scores

**DOI:** 10.1186/s12872-023-03628-7

**Published:** 2023-12-13

**Authors:** Zhengli Luo, Yuan Feng, Dan Luo, Shiyang Li, Kaiyi Xiao, Hongmei Shen, Qiang Hu

**Affiliations:** 1 Emergency Department, Panzhihua Central Hospital, No. 34 Yikang Street, Middle Section of Panzhihua Avenue, Panzhihua, 617000 China; 2 Division of Cardiology, Panzhihua Central Hospital, Panzhihua, China; 3 Information Center, Panzhihua Central Hospital, Panzhihua, China

**Keywords:** Thrombolysis in myocardial infarction score, ST-segment myocardial infarction, In-hospital survival, Favorable factors, Emergency percutaneous coronary intervention, Receiver operating characteristics curve, Logistic regression analysis

## Abstract

**Objective:**

ST-segment myocardial infarction (STEMI) is a time-sensitive emergency. This study screened the favorable factors for the survival of STEMI patients with medium- and high-risk thrombolysis in myocardial infarction (TIMI) scores.

**Methods:**

According to the TIMI scores at admission, 433 STEMI patients were retrospectively and consecutively selected and allocated into low-/medium-/high-risk groups, with their general information/blood routine/biochemical indicators/coagulation indicators documented. The factors influencing the in-hospital survival of STEMI patients were analyzed using univariate and multivariate logistic regression analyses. Moreover, the predictive value of favorable factors was analyzed by receiver operating characteristics (ROC) curve, and patients were assigned into high/low level groups based on the cut-off value of these factors, with their in-hospital survival rates compared.

**Results:**

The in-hospital survival rate of the medium-/high-risk groups was lower than that of the low-risk group. Emergency percutaneous coronary intervention (PCI), lymphocyte (LYM), total protein (TP), albumin (ALB), and sodium (Na) were independent favorable factors for in-hospital survival in the medium-/high-risk groups. Besides, LYM > 1.275 × 10^9^/L, TP > 60.25 g/L, ALB > 34.55 g/L, and Na > 137.9 mmo1/L had auxiliary predictive value for the survival of STEMI patients with medium-/high-risk TIMI scores. Patients with high levels of LYM, TP, ALB, and Na exhibited higher in-hospital survival rates than patients with low levels.

**Conclusion:**

For STEMI patients with medium- and high-risk TIMI scores, accepting emergency PCI and normal levels of LYM, TP, ALB, and Na were more conducive to in-hospital survival.

**Supplementary Information:**

The online version contains supplementary material available at 10.1186/s12872-023-03628-7.

## Introduction

Coronary artery disease (CAD), mostly caused by atherosclerosis of arteries that supply blood to the heart and manifested by narrowing or blockage of narrowing or blockage of coronary arteries, remains one of the leading causes of death in the majority of developed countries [1, 2]. Currently, extensive studies have explored the related factors of CAD and its severity. As reported, several blood flow reserve ratios, such as the monocyte/high-density lipoprotein cholesterol ratio, neutrophil/lymphocyte ratio, platelet/lymphocyte ratio, and lymphocyte/monocyte ratio, have been identified as potential indicators for assessing the severity of CAD (DOI: https://doi.org/10.5152/EurJTher.2022.22037). In addition, the ORBIT risk score is an independent predictor of massive bleeding in patients with acute coronary syndrome [3]. Among CADs, acute myocardial infarction (AMI) is the most perilous and has emerged as a type of high-risk and thrombotic cardiovascular disease that posed a serious hazard to individuals' well-being. Myocardial infarction (MI), generally known as “heart attack”, resulted from reduced or complete cessation of blood flow to parts of the myocardium, thereby leading to damage to the heart muscle [4]. As a common type of MI, the incidence of ST-segment elevation myocardial infarction (STEMI) is 0.05 to 0.15% per year [5]. The pathophysiologic basis for STEMI is the occurrence of severe or complete blockage of the culprit coronary artery due to thrombus formation, which disrupts the intracoronary plaque [6]. A previous study reported that the in-hospital mortality rate of STEMI patients in China was roughly 7% to 9%, with the 1-year mortality rate reaching as high as 28% [7]. As a consequence of transmural ischemia of the myocardium, percutaneous coronary intervention (PCI) is recommended as the first-line therapy for STEMI [8]. Additionally, prompt identification of factors connected with improved survival outcomes in STEMI patients is essential for optimizing their care and guiding management decisions.

In the past 20 years, Several risk scoring models have been developed based on various risk factors, which can help clinicians stratify patients with risk factors and predict the mortality risk and/or the incidence of adverse events [9]. The Global Registry of Acute Coronary Events score, the Thrombolysis in Myocardial Infarction (TIMI) score, and the HEART score are the three most frequently used scoring models for CAD, among which the TIMI score can be utilized for differentiating STEMI patients at different risk levels [10, 11]. The TIMI score is a widely recognized and validated risk stratification tool for evaluating the severity and prognosis of patients with MI [12]. The TIMI score incorporates diverse clinical parameters and helps categorize patients into distinct risk groups, allowing healthcare providers to tailor treatment strategies accordingly. In this study, we aimed to screen the favorable factors for the survival of STEMI patients with medium- and high-risk TIMI scores and to lay a foundation for clinical management strategies formulated by medical practitioners in a clinical setting. For this objective, we analyzed the clinical baseline data as well as blood routine, blood biochemistry, and coagulation data.

## Materials and methods

### Ethics statement

All subjects were fully informed of the study objective and signed an informed consent form. This study complied with the Declaration of Helsinki and was approved by the Ethics Committee of Panzhihua Central Hospital.

### Sample size estimation

The sample size estimation software G * Power 3.0.10, developed by the University of Dusseldorf (Germany), was used to estimate the sample size. The corresponding parameter settings were: Power = 0.95, α = 0.05, Effect size = 0.25, Number of groups = 3. ANOVA: Fixed effects, omnibus, one-way in F tests were selected, and the analysis showed a minimum total sample size of 252 cases (Supplementary Fig. 1).

### Study subjects

A total of 777 STEMI patients admitted to Panzhihua Central Hospital from December 2018 to April 2022 were retrospectively and consecutively selected, of which 236 had incomplete clinical data, and 108 were excluded from this study as per the inclusion and exclusion criteria, and eventually 433 patients were enrolled as subjects. In line with the TIMI score at admission, the subjects were separated into the low-risk group (*n* = 195, 0–3 points), the medium-risk group (*n* = 165, 4–6 points), and the high-risk group (*n* = 73, 7–14 points).

Inclusion criteria were as follows: 1) adults over 18 years old; 2) conform to the diagnostic criteria for STEMI.

Exclusion criteria were as below: 1) incomplete clinical data; 2) a history of malignant tumors, recent chemotherapy, or organ transplantation; 3) recent severe bleeding, infection, liver and kidney failure; 4) autoimmune system diseases; 5) acute trauma history or acute blood loss; 6) elevated myocardial injury markers resulting from aortic dissection and patients who were transferred.

### Diagnostic criteria

STEMI was diagnosed in accordance with the Guidelines for the Diagnosis and Treatment of Acute ST-segment Elevation Myocardial Infarction (2019) and the World Health Organization’s diagnostic criteria, based on the chest pain and related symptoms, the dynamic changes of ST-T in the electrocardiogram (ECG), and the dynamic alterations of serum myocardial injury markers of the patients. Specifically, patients met two of the following criteria: (1) chest pain lasting over 30 min; (2) ST segment elevation and continuous change observed in ECG; (3) obviously increased intraserum troponin and higher than the normal value and (or) no less than a 2-time level of CK and (or) CK-MB of the normal value.

### TIMI scoring

In some large-scale clinical trials based on the TIMI risk score, age, systolic blood pressure (SBP), and heart rate are the factors that can independently play a predictive role and have the most dominant impact in terms of their ability to predict the poor clinical prognosis of STEMI patients [13]. The specific process of performing TIMI scoring for patients with STEMI was as follows: 1. different ages were corresponding to varying ratings, with the age groups under 65, 65–74, and over 75 years scoring 0, 2, and 3 points, respectively; 2. SBP not reaching 100 mmHg was recorded as 3 points; 3. the heart rate ≥ 100 beats/min, 2 points; 4. Killip rating II-IV, 2 points; 5. ST-segment elevation or left bundle branch block, 1 point; 6. diabetes, hypertension, or angina pectoris, 1 point; 7. body weight < 67 kg, 1 point; 8. treatment time from onset > 4 h, 1 point.

### Observation indicators

The general data of the patients were recorded, including age, sex, height, blood pressure and heart rate at admission, time of onset, location of myocardial infarction, and whether emergency PCI was performed or not. The following collected indicators included: 1) first blood routine on admission [white blood cell (WBC) count, neutrophil (NEUT) count, lymphocyte (LYM) count, mononuclear cell (MNC) count, lymphocyte ratio (LYMR), mononuclear cell ratio (MNCR), eosinophil ratio (EOSR), basophil ratio (BASR), hemoglobin (Hb), erythrocyte count (ERY), hematocrit (HCT), mean corpuscular volume (MCV), mean hemoglobin (MHb), mean corpuscular hemoglobin concentration (MCHC), red blood cell distribution width-standard deviation (SD), red blood cell distribution width coefficient of variation (CV), platelet (PLT) count, mean platelet volume (MPV), platelet distribution width (PDW), large platelets ratio (LPR), platelet crit (PCT)]; 2) biochemical indicators [alanine aminotransferase (ALT), aspartate aminotransferase (AST), γ-glutamyl transpeptidase (GGT), serum creatinine (SCr), troponin I (TnI), total protein (TP), albumin (ALB), globulin (GLB), albumin/globulin ratio (A/G ratio), total cholesterol (TC), triglyceride (TG), high-density lipoprotein (HDL), low-density lipoprotein (LDL), blood urea nitrogen, blood potassium (K), blood sodium (Na), blood calcium (Ca), blood sugar]; 3) coagulation indicators [prothrombin time (PT), prothrombin activity (PtA)]. The in-hospital survival and death of all subjects were counted.

### Statistical analysis

Statistical analysis and graphing were performed using SPSS 21.0 (IBM Corp. Armonk, NY, USA) and GraphPad Prism 8.01 (GraphPad Software, San Diego, CA, USA) software. The Shapiro–Wilk test was employed for the normal distribution, and the measurement data of the normal distribution were expressed as mean ± SD and tested by an independent sample *t*-test or one-way analysis of variance (ANOVA). Subsequent post hoc tests were conducted using Tukey’s multiple comparisons test. The non-normally distributed measurement data were represented by quartiles [median (minimum, maximum)] and tested by the Mann–Whitney U test or the Kruskal–Wallis H test. Categorical variables were presented using the number of cases or percentages and analyzed by the Chi-square test. The factors influencing the in-hospital survival of STEMI patients were analyzed by univariate and multivariate logistic regression analyses. The receiver operating characteristics (ROC) curve was used to analyze the predictive value of favorable factors for the in-hospital survival of STEMI patients with medium- and high-risk TIMI scores, with the area under the curve (AUC) calculated. AUC = 1 was the most ideal predictor, and AUC < 0.5 was regarded to be of no predictive value. Stastistical significance was defined as a value of *P* < 0.05.

## Results

### Comparisons of clinical data

We retrospectively selected 433 STEMI patients who attended Panzhihua Central Hospital from December 2018 to April 2022 as the study subjects. According to the TIMI score at admission, the subjects were distributed into the low-risk group (*n* = 195), the medium-risk group (*n* = 165), and the high-risk group (*n* = 73), with their clinical data collected and analyzed. The three groups had no statistical differences in terms of NEUT, MNC, LYMR, MNCR, EOSR, MHb, PLT, MPV, PDW, LPR, PCT, ALT, GGT, TnI, TP, HDL, K, Na, Ca, or blood sugar (all *P* > 0.05) (Supplementary Table 1). Relative to the low-risk group, the medium- and high-risk groups were noticeably different in age, sex, weight, diastolic blood pressure, time of onset, myocardial infarction site, Hb, ERY, HCT, MCHC, SD, ALB, A/G, TC, TG, LDL, blood urea nitrogen, PT, and PtA (all *P* < 0.05) (Table 1). In respect of emergency PCI, SBP, heart rate, WBC, LYM, BASR, MCV, CV, AST, SCr, and K, there were marked distinctions between the high-risk and low-risk groups (all *P* < 0.05), while no statistical differences were found between the medium-risk and low-risk groups (all *P* > 0.05) (Table 1).

**Table 1:** Comparisons of clinical data of three groups of STEMI patients

	Low-risk group (*n* = 195)	Medium-risk group (*n* = 165)	High-risk group (*n* = 73)	*Pa*	*Pb*
**General data**					
Age (Year)	53.00 (31.00, 77.00)	66.00 (37.00, 88.00)	73.00 (28.00, 90.00)	< 0.0001	< 0.0001
**Gender**					
Male (n, %)	175 (89.74%)	132 (80.00%)	46 (63.01%)	0.0093	< 0.0001
Female (n, %)	20 (10.26%)	33 (20.00%)	27 (36.99%)		
Weight (kg)	70.00 (47.00, 105.0)	62.00 (32.00, 91.00)	62.00 (40.00, 86.00)	< 0.0001	< 0.0001
Emergency PCI (n, %)	116 (59.49%)	74 (44.85%)	23 (31.51%)	0.0056	< 0.0001
SBP (mm Hg)	128.0 (100.0, 225.0)	125.0 (82.00, 199.0)	107.0 (60.00, 176.0)	0.2980	< 0.0001
DBP (mm Hg)	81.00 (59.00, 133.0)	76.00 (47.00, 123.0)	67.00 (46.00, 125.0)	0.0033	< 0.0001
Heart rate (beats/min)	77.00 (45.00, 106.0)	78.00 (39.00, 143.0)	85.00 (40.00, 151.0)	0.4267	< 0.0001
Time of onset (h)	7.00 (0.50, 480.0)	10.00 (0.50, 720.0)	23.00 (1.00, 720.0)	0.0304	< 0.0001
**Myocardial infarction site**				0.0063	< 0.0001
Anterior wall (n)	80	93	53		
Inferior wall (n)	111	71	19		
Lateral wall (n)	4	0	1		
Posterior wall (n)	0	1	0		
**Blood routine**					
WBC (× 10^9^/L)	10.89 (4.27, 19.99)	10.17 (3.74, 22.04)	8.75 (1.37, 28.09)	0.1745	0.0014
LYM (× 10^9^/L)	1.65 (0.50, 6.71)	1.48 (0.39,4.38)	1.42 (00.32, 3.88)	0.0581	0.0043
BASR (%)	0.30 (0.00, 1.30)	0.30 (0.00, 1.60)	0.40 (0.10, 17.96)	0.4039	0.0450
Hb (g/L)	148.0 (92.00, 203.0)	140.0 (52.00, 176.0)	130.0 (60.00, 178.0)	< 0.0001	< 0.0001
ERY (× 10^12^/L)	4.83 (2.86, 6.37)	4.56 (2.30, 5.76)	4.34 (2.19, 5.67)	< 0.0001	< 0.0001
HCT (%)	44.70 (29.10, 58.50)	42.40 (16.50, 52.80)	39.90 (19.50, 52.60)	< 0.0001	< 0.0001
MCV (fL)	91.70 (77.50, 104.0)	92.90 (62.50, 107.7)	93.40 (76.40, 105.2)	0.0643	0.0079
MCHC (g/L)	334.10 ± 10.94	330.70 ± 11.32	325.40 ± 11.27	0.0069	< 0.0001
RDW (SD, fL)	43.70 (36.50, 58.50)	44.80 (36.90, 62.80)	46.50 (35.40, 58.90)	0.0202	< 0.0001
CV (%)	13.00 (10.80, 17.80)	13.20 (11.70, 17.80)	13.50 (11.80, 17.20)	0.1118	0.0023
**Biochemical indicators**					
AST (U/L)	82 (0, 690)	84 (0, 1299)	47 (0, 511)	> 0.9999	0.0312
SCr (μmol/L)	77 (44, 222)	80 (41, 161)	90 (47, 391)	0.7265	< 0.0001
ALB (g/L)	40.90 (27.40, 54.30)	39.90 (25.00, 47.30)	37.70 (26.40, 46.70)	0.0004	< 0.0001
GLB (g/L)	23.21 ± 3.439	24.69 ± 4.075	25.51 ± 4.472	0.0007	< 0.0001
A/G	1.77 (1.09, 2.86)	1.59 (0.83, 2.48)	1.43 (0.88, 2.48)	< 0.0001	< 0.0001
TC (mmo1/L)	4.32 (2.38, 8.64)	4.07 (2.20, 7.58)	4.00 (1.93, 8.44)	0.0226	0.0158
TG (mmo1/L)	1.63 (0.35, 12.20)	1.33 (0.34, 6.20)	1.27 (0.43, 7.80)	0.0022	0.0085
LDL (mmo1/L)	2.90 (1.05, 6.35)	2.59 (0.95, 6.13)	2.51 (0.78, 6.75)	0.0062	0.0169
Blood urea nitrogen (mmo1/L)	4.70 (1.40, 14.76)	5.16 (1.40, 18.00)	6.60 (2.60, 25.96)	0.0273	< 0.0001
K	3.92 (2.67, 7.90)	4.02 (2.68, 4.99)	4.04 (3.15, 5.77)	0.6244	0.0407
**Coagulation indicators**					
PT (s)	11.6 (9.6, 21.4)	11.8 (9.9, 20.1)	12.1 (10.7, 20.4)	0.0372	0.0003
PtA (%)	94.0 (36.0, 138.0)	90.0 (40.0, 135.0)	85.0 (37.0, 117.0)	0.0099	0.0003

*Note*: *SBP* Systolic blood pressure, *DBP* Diastolic blood pressure, *WBC* White blood cell, *LYM* Lymphocyte, *BASR* Basophil ratio, *Hb* Hemoglobin, *ERY* Erythrocyte, *HCT* Hematocrit, *MCV* Mean corpuscular volume, *MCHC* Mean corpuscular hemoglobin concentration, *RDW* Red blood cell distribution width, *CV* Coefficient of variation, *AST* Aspartate aminotransferase, *SCr* Serum creatinine, *ALB* Albumin, *GLB* Globulin, *TC* Total cholesterol, *TG* Triglyceride, *LDL* Low density lipoprotein, *K* Potassium, *PT* Prothrombin time, *PtA* Prothrombin activity. The Shapiro–Wilk test was used to test the normal distribution, and the measurement data of the normal distribution were expressed as the mean ± SD, and analyzed by one-way ANOVA, followed by Tukey’s multiple comparisons test. The non-normally distributed measurement data were represented by quartiles [median value (minimum value, maximum value)], and analyzed by the Kruskal–Wallis H test. Categorical variables were expressed using the number of cases or percentages, and analyzed by Chi-square test. *Pa*: the medium-risk group vs. the low-risk group; *Pb*: the high-risk group vs. the low-risk group. When TnI > 25 ng/mL, the patient had severe myocardial injury

### Comparison of in-hospital survival rate

Further, we statistically analyzed the in-hospital survival rate in the low-, medium-, and high-risk groups and we discovered that the in-hospital survival rate was 97.95% in the low-risk group, 93.94% in the medium-risk group, and 82.19% in the high-risk group. Patients in the medium- and high-risk groups demonstrated obviously lower in-hospital survival rates than patients in the low-risk group (all *P* < 0.05) (Table 2).

**Table 2:** Comparison of in-hospital survival rate of STEMI patients

	Low-risk group (*n* = 195)	Medium-risk group (*n* = 165)	High-risk group (*n* = 73)	*Pa*	*Pb*
In-hospital survival rate	97.95%	93.94%	82.19%	0.0499	< 0.0001

*Note*: Data were expressed as percentages, and analyzed by Chi-square test. *Pa*: the medium-risk group vs. the low-risk group; *Pb*: the high-risk group vs. the low-risk group

### Comparative analysis of clinical data between surviving and dead STEMI patients with medium- and high-risk TIMI scores

According to the occurrence of in-hospital death, the STEMI patients in the medium- and high-risk groups were subdivided into the survival group (*n* = 215) and the death group (*n* = 23), with their clinical data compared. As illustrated in Table 3, the two groups statistically varied in weight, emergency PCI, heart rate, NEUT, LYM, LYMR, Hb, HCT, MHb, MCHC, CV, ALT, GGT, SCr, TP, ALB, TC, blood urea nitrogen, Na, blood glucose, and PT (all *P* < 0.05), while exhibiting no salient distinctions in other clinical data (all *P* > 0.05) (Supplementary Table 2).

**Table 3:** Comparative analysis of clinical data between surviving and dead STEMI patients with medium- and high-risk TIMI scores

	Survival group (*n* = 215)	Death group (*n* = 23)	*P*
**General data**			
Weight (kg)	62.14 ± 10.080	66.78 ± 9.625	0.0362
Emergency PCI (n, %)	94 (43.72%)	3 (13.04%)	0.0044
Heart rate (beats/min)	79 (39, 151)	93 (48, 136)	0.0009
**Hematology**			
NEUT (× 10^9^/L)	7.34 (1.40, 22.85)	10.15 (2.62, 19.55)	0.0428
LYM (× 10^9^/L)	1.48 (0.37, 4.38)	0.97 (0.32, 1.87)	< 0.0001
LYMR (%)	15.90 (2.70, 54.60)	9.66 (1.37, 33.10)	0.0014
Hb (g/L)	137.00 (60.00, 178.00)	123.00 (52.00, 167.00)	0.0055
HCT (%)	41.80 (19.50, 52.80)	38.30 (16.50, 50.10)	0.0359
MHb (pg)	30.90 (23.00, 36.20)	29.70 (18.60, 33.00)	0.0062
MCHC (g/L)	330.00 (302.00, 365.00)	317.00 (298.00, 342.00)	0.0002
CV of RDW (%)	13.20 (11.70, 17.50)	13.70 (12.00, 17.80)	0.0188
**Biochemical indicators**			
ALT (U/L)	30 (5, 1450)	41 (12, 270)	0.0243
GGT (U/L)	31 (0, 645)	50 (0, 268)	0.0298
SCr (μmol/L)	81 (41, 391)	100 (55, 303)	0.0012
TP (g/L)	64.10 ± 5.694	59.91 ± 8.609	0.0018
ALB (g/L)	39.30 (26.40, 47.20)	36.10 (25.00, 47.30)	0.0262
TC (mmo1/L)	4.06 (2.20, 8.41)	3.26 (1.93, 8.44)	0.0037
LDL (mmo1/L)	2.60 (0.95, 6.43)	1.88 (0.78, 6.75)	0.0070
Blood urea nitrogen (mmo1/L)	5.27 (1.40, 25.96)	7.50 (1.80, 18.00)	0.0007
Na (mmo1/L)	139.9 (127.2, 154.2)	137.5 (120.1, 149.1)	0.0293
Blood sugar (mmol/L)	6.36 (3.23, 28.28)	11.71 (4.06, 30.44)	0.0003
**Coagulation indicator**			
PT (s)	11.90 (9.90, 20.40)	12.80 (10.40, 15.90)	0.0150

*Note*: *NEUT* Neutrophil, *LYM* Lymphocyte, *LYMR* Lymphocyte ratio, *MHb* Mean hemoglobin, *MCHC* Mean corpuscular hemoglobin concentration, *RDW* Red blood cell distribution width, *CV* Coefficient of variation, *ALT* Alanine aminotransferase, *GGT* Gamma-glutamyl transpeptidase, *SCr* Serum creatinine, *TP* Total protein, *ALB* Albumin, *TC* Total cholesterol, *LDL* Low density lipoprotein, *Na* sodium, *PT* Prothrombin time. The Shapiro–Wilk test was used to test the normal distribution, and the measurement data of normal distribution were expressed as mean ± SD, and analyzed by independent-sample *t* test. The non-normally distributed measurement data were represented by quartiles, i.e., the median value (minimum value, maximum value), and analyzed by Mann–Whitney U test. Categorical variables were expressed by percentages, and analyzed by Chi-square test. When TnI > 25 ng/mL, the patient had severe myocardial injury

### Logistic regression analysis of favorable factors for in-hospital survival in STEMI patients with medium- and high-risk TIMI scores

According to the analysis results in Table 3, clinical data (body weight, emergency PCI, heart rate, NEUT, LYM, LYMR, Hb, HCT, MHb, MCHC, CV, ALT, GGT, SCr, TP, ALB, TC, LDL, blood urea nitrogen, Na, blood glucose, and PT) with notable differences between patients in the surviving and death groups were included in the univariate logistic regression analysis. The analysis revealed that body weight, heart rate, NEUT, CV, SCr, blood urea nitrogen, and blood glucose were risk factors for in-hospital survival in the medium- and high-risk groups; emergency PCI, LYM, LYMR, Hb, HCT, MHb, MCHC, TP, ALB, TC, LDL, and Na were favorable factors for in-hospital survival in the medium- and high-risk groups (all *P* < 0.05). As reflected by the results of the univariate regression analysis, favorable factors with *P* < 0.05 for in-hospital survival of STEMI patients with medium- and high-risk TIMI scores were enrolled in the multivariate logistic regression analysis as independent variables. Beyond that, we found that emergency PCI, LYM, TP, ALB, and Na were independent favorable factors for in-hospital survival in STEMI patients with medium- and high-risk TIMI scores (all *P* < 0.05) (Table 4).

**Table 4:** Logistic regression analysis of favorable factors for in-hospital survival in STEMI patients with medium- and high-risk TIMI scores

	Univariate logistic regression analysis				Multivariate logistic regression analysis			
	B	OR	*P*	95% CI	B	OR	*P*	95% CI
Weight	-0.47	0.954	0.038	0.913–0.997				
Emergency PCI	1.645	5.179	0.010	1.494–17.952	4.008	55.016	0.001	4.868–621.757
Heart rate	-0.038	0.962	0.001	0.942–0.984				
NEUT	-0.143	0.867	0.006	0.783–0.960				
LYM	1.781	5.937	0.000	2.219–15.887	1.576	4.834	0.046	1.027–22.749
LYMR	0.096	1.100	0.004	1.031–1.174	0.021	1.022	0.705	0.915–1.141
Hb	0.034	1.035	0.000	1.016–1.055	-0.134	0.875	0.775	0.349–2.192
HCT	0.101	1.106	0.002	1.037–1.180	0.453	1.574	0.765	0.081–30.723
MHb	0.313	1.368	0.000	1.159–1.615	0.134	1.143	0.357	0.860–1.518
MCHC	0.088	1.092	0.000	1.047–1.139	0.133	1.143	0.486	0.785–1.664
CV	-0.473	0.623	0.005	0.449–0.865				
ALT	0.005	1.005	0.075	0.999–1.011				
GGT	-0.004	0.996	0.081	0.992–1.000				
SCr	-0.014	0.986	0.011	0.976–0.997				
TP	0.100	1.105	0.003	1.035–1.179	0.159	1.173	0.037	**1.010–1.362**
ALB	0.150	1.161	0.003	1.053–1.281	0.242	0.785	0.047	**0.619–0.997**
TC	0.583	1.792	0.019	1.099–2.922	1.004	2.728	0.240	0.511–14.569
LDL	0.562	1.753	0.043	1.017–3.024	-0.856	0.425	0.379	0.063–2.854
Blood urea nitrogen	-0.182	0.834	0.002	0.744–0.935				
Na	0.160	1.174	0.002	1.060–1.299	0.156	1.169	0.041	**1.006–1.358**
Blood sugar	-0.157	0.855	0.000	0.795–0.919				
PT	-0.200	0.819	0.076	0.656–1.021				

*Note*: *NEUT* Neutrophil, *LYM* Lymphocyte, *LYMR* Lymphocyte ratio, *Hb* Hemoglobin, *HCT* Hematocrit, *MHb* mean hemoglobin, *MCHC* Mean corpuscular hemoglobin concentration, *CV* Coefficient of variation, *ALT* Alanine aminotransferase, *GGT* Gamma-glutamyl transpeptidase, *SCr* Serum creatinine, *TP* Total protein, *ALB* Albumin, *TC* Total cholesterol, *LDL* Low density lipoprotein, *Na* sodium, *PT* Prothrombin time

### ROC curve analysis of favorable factors for in-hospital survival in STEMI patients with medium- and high-risk TIMI scores

**Fig. 1:**
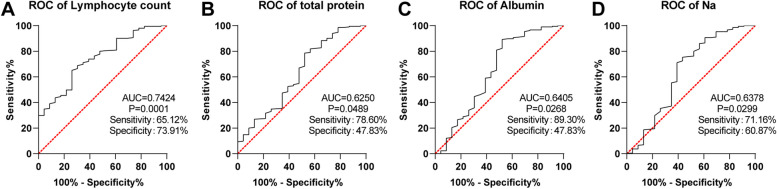
ROC curve analysis of favorable factors for in-hospital survival in STEMI patients with medium- and high-risk TIMI scores

In the quest to further explore the predictive value of LYM, TP, ALB, and Na on the in-hospital survival of STEMI patients with medium- and high-risk TIMI scores, we used LYM, TP, ALB, and Na as independent variables and in-hospital survival as the dependent variable to plot ROC curves. LYM (AUC = 0.7424, *P* = 0.0001, sensitivity 65.12%, specificity 73.91%, cut-off value 1.275), TP (AUC = 0.6250, *P* = 0.0489, sensitivity 78.60%, specificity 47.83%, cut-off value 60.25), ALB (AUC = 0.6405, *P* = 0.0268, sensitivity 89.30%, specificity 47.83%, cut-off value 34.55), and Na (AUC = 0.6378, *P* = 0.0299, sensitivity 71.16%, specificity 60.87%, cut-off value 137.9) had certain auxiliary predictive value for the in-hospital survival of STEMI patients with medium- and high-risk TIMI scores (Fig. 1A-D).

### Cut-off value analysis

In the light of the ROC analysis, the cut-off value was 1.275 for LYM, 60.25 for TP, 34.55 for ALB, and 137.9 for Na. We further classified STEMI patients with medium- and high-risk TIMI scores into the high-level group (LYM, TP, ALB, Na) and the low-level group (LYM, TP, ALB, Na), and statistically analyzed their in-hospital survival rates. The results elicited that the in-hospital survival rate of the high-level group was apparently higher than that of the low-level group (*P* < 0.05) (Table 5).

**Table 5:** Cut-off value analysis

	In-hospital survival (n)	Death	In-hospital survival rate	*P*
LYM				0.0003
High-level group (*n* = 146)	140	6	95.89%	
Low-level group (*n* = 92)	75	17	81.52%	
TP				0.0048
High-level group (*n* = 181)	169	12	93.37%	
Low-level group (*n* = 57)	46	11	80.70%	
ALB				< 0.0001
High-level group (*n* = 204)	192	12	94.12%	
Low-level group (*n* = 34)	23	11	67.65%	
Na				0.0021
High-level group (*n* = 161)	152	9	94.41%	
Low-level group (*n* = 77)	63	14	81.82%	

*Note*: Categorical variables were expressed by percentages, and analyzed by Chi-square test

*LYM* Lymphocyte, *TP* Total protein, *ALB* Albumin, *Na* sodium

## Discussion

STEMI remains the leading cause of mortality around the world, but mortality has reduced owing to various preventive measures and the development of early diagnosis and treatment [14, 15]. Pathologically, STEMI is underpinned by the formation of thromboses, which disrupts the intracoronary plaque and leads to near-total or total occlusion of the culprit coronary artery [6]. The TIMI score is frequently employed for the assessment of thrombus burden, serving as a crucial clinical instrument in the evaluation and treatment of patients with MI [12]. In this study, we assigned the subjects into low-risk, medium-risk, and high-risk groups according to their TIMI scores at admission, analyzed and compared the in-hospital survival rate of each group, and found that emergency PCI and LYM, TP, ALB, and Na at a normal level were more conducive to the in-hospital survival of patients, which were independent favorable factors for the in-hospital survival of high-risk STEMI patients with TIMI scores.

The TIMI score is widely utilized for distinguishing STEMI patients of different risk levels [10, 11]. Our findings revealed prominent discrepancies in the in-hospital survival rates between the low-risk group and the medium- and high-risk groups. In particular, patients in the medium and high-risk groups exhibited an apparently lower survival rate than patients in the low-risk group. This discrepancy underscores the importance of risk stratification using the TIMI score, as it allows for early identification of higher-risk patients and the implementation of appropriate interventions to enhance their outcomes [16–18]. Also, another study has demonstrated that the combination of SYNTAX score II (SS-II) and TIMI can more accurately predict individual in-hospital and long-term mortality in STEMI patients [18]. What’s more, Anggoro Budi Hartopo et al. have reported that a high TIMI risk score is related to a higher mean endothelin-1 level and endothelin-1: endothelin-3 ratio, which provides a prognostic value of 1-year mortality in STEMI patients [17]. In spite of its usefulness, it is worth noting that the TIMI score is not the sole determinant of treatment decisions. However, each patient’s condition is unique, and the development of treatment for different patients requires a full understanding of the patient’s medical history, symptoms, signs and other information, adherence to the principle of comprehensiveness, combination of relevant examination results and laboratory data, and comprehensive consideration of a number of factors. Furthermore, for proper understanding, the score needs to be placed in the context of applicable clinical guidelines and healthcare facility resources. Therefore, on this basis, we further explored the favorable factors for in-hospital survival in patients of medium- and high-risk STEMI, and discovered that emergency PCI, LYM, TP, ALB, and Na levels emerged as independent favorable factors for the in-hospital survival of patients in the medium and high-risk groups. Those findings have important clinical implications. TIMI scores combined with various clinical parameters could help health care professionals divide patients into different risk groups, adjust treatment strategies accordingly, and diminish the in-hospital mortality of STEMI patients. In a cohort study conducted in Turkey, TIMI bleeding scores along with Determining a patient’s Predicting Bleeding Complication in Patients Undergoing Stent Implantation and Subsequent Dual Antiplatelet Therapy (PRECISE-DAPT) scores were used to conduct bleeding classification and evaluate bleeding risk in patients with acute coronary syndrome or those undergoing PCI [19]. Consistent with our finding, emergency PCI, a procedure used to restore blood flow to the blocked coronary arteries, has been identified as a significant favorable factor in patients with STEMI [20]. Beyond that, the optimal administration of antithrombotic therapy in patients diagnosed with STEMI is of utmost significance. Evidence suggests that monotherapy with an oral P2Y12 inhibitor after short DAPT regimens is related to myocardial infarction, all-cause mortality, and stroke risks in patients receiving complex PCI [21]. Nevertheless, since the intricate nature of coronary artery anatomy and the specific clinical circumstances of patients, there is a possibility of encountering adverse events following PCI, including inadequate revascularization [22] and hemorrhagic events [23]. It is also noteworthy that timely reperfusion therapy plays a crucial role in mitigating myocardial damage and raising survival in STEMI patients, especially for those at higher risk [24]. LYM, TP, ALB, and Na levels were observed to independently influence the survival rates and possessed certain auxiliary predictive value for the in-hospital survival of STEMI patients with medium- and high-risk TIMI scores. Except for this, their in-hospital survival and those parameters reflected the patient’s immune status and electrolyte balance, underlining the importance of addressing immune function, nutritional support, and electrolyte imbalances in the matter of treating medium- and high-risk STEMI patients. Moreover, medium- to high-risk TIMI-socred patients with high levels of LYM, TP, ALB, and Na exhibited an evidently higher in-hospital survival rate. Consistently, previous evidence emphasized the important role of immune function in the progression of MI with regard to reducing the inflammatory response [25]. The study conducted by Ji Z et al. showed that neutrophil-to-lymphocyte ratio (NLR) had a certain predictive ability for in-hospital death in STEMI patients [26]. NLR and platelet-to-lymphocyte ratio (PLR) in peripheral blood are directly associated with the occurrence of significant adverse events in hospitalization following STEMI [27]. NLR serves as a prognostic indicator for both hospitalization rates and long-term outcomes in patients with STEMI following PCI, but large randomized clinical trials are required to further confirm this [28]. Furthermore, there is a negative correlation observed between serum albumin (ALB)-to-creatinine ratio (sACR) and in-hospital mortality following PCI in patients diagnosed with STEMI, and higher level of ALB favors post-PCI in-hospital survival in patients with STEMI [29]. Patients with STEMI who have low serum ALB level are more likely to experience serious complications while hospitalized, suggesting that this factor contributes to the prothrombotic phenotype of patients [30]. Moreover, among patients with STEMI treated with PCI, those with lower serum sodium level on admission have a higher risk of poorer in-hospital clinical prognosis, and patients with hyponatremia have a higher mortality rate during long-term follow-up [31]. However, there are certain limitations in terms of sample size, statistical analysis methods, and inclusion indicators. In this study, we included a relatively larger sample size (433 STEMI patients) and used a variety of statistical analysis methods, such as single-factor difference analysis, single-factor and multi-factor logistic regression analysis, and ROC curve analysis. Based on the TIMI score, dozens of specific case indicators in terms of clinical general data, blood routine, biochemical indicators, and coagulation indicators were used to conduct a more in-depth and comprehensive analysis of the favorable factors for in-hospital survival of STEMI patients of medium and high risk. It was found that emergency PCI, LYM, TP, ALB, and Na were independent favorable factors for in-hospital survival in high-risk STEMI patients in TIMI scores. The results were more convincing and provided valuable theoretical references for clinical risk assessment and treatment strategy formulation.

Although our study found some clinically meaningful results, it is important to acknowledge certain limitations of our study. Firstly, the retrospective nature of the study design might bring inherent biases and impede the establishment of causal relationships between indicators and adverse outcomes. Secondly, the study was carried out at a single center, perhaps influencing the generalizability of the findings to alternative healthcare settings. Additionally, other potential factors that could potentially impact survival outcomes, such as comorbidities and specific treatment regimens, were not thoroughly examined in this study and probably need further investigation in future studies.

In conclusion, our study highlighted the conspicuous differences in in-hospital survival rates among STEMI patients with varying TIMI risk scores. The identification of emergency PCI and normal levels of LYM, TP, ALB, and Na as independent favorable factors for survival highlights their crucial role in improving outcomes for medium- and high-risk patients. These findings provide valuable insights for risk assessment and treatment strategies, help healthcare professionals identify patients at higher risk of poor outcomes, and guide the selection of appropriate treatment strategies, ultimately enhancing patient outcomes and lowering morbidity and mortality associated with this life-threatening condition.

### Supplementary Information

**Additional file 1:** **Supplementary Figure 1.** Sample size estimation.

**Additional file 2:** **Supplementary Table 1.** Comparisons of clinical data of three groups of STEMI patients. **Supplementary Table 2.** Comparative analysis of clinical data between surviving and dead STEMI patients with medium- and high-risk TIMI scores.

## Data Availability

All data generated or analysed during this study are included in this article. Further enquiries can be directed to the corresponding author.
