# Supplementary figures and images for: Favorable factors for the survival of ST-segment elevation myocardial infarction patients with medium- and high-risk thrombolysis in myocardial infarction scores
Source: BMC Cardiovasc Disord. 2023 Dec 13;23:614. doi: 10.1186/s12872-023-03628-7 (PMC10720153; doi:10.1186/s12872-023-03628-7)

**Supplementary Figure 1** Sample size estimation


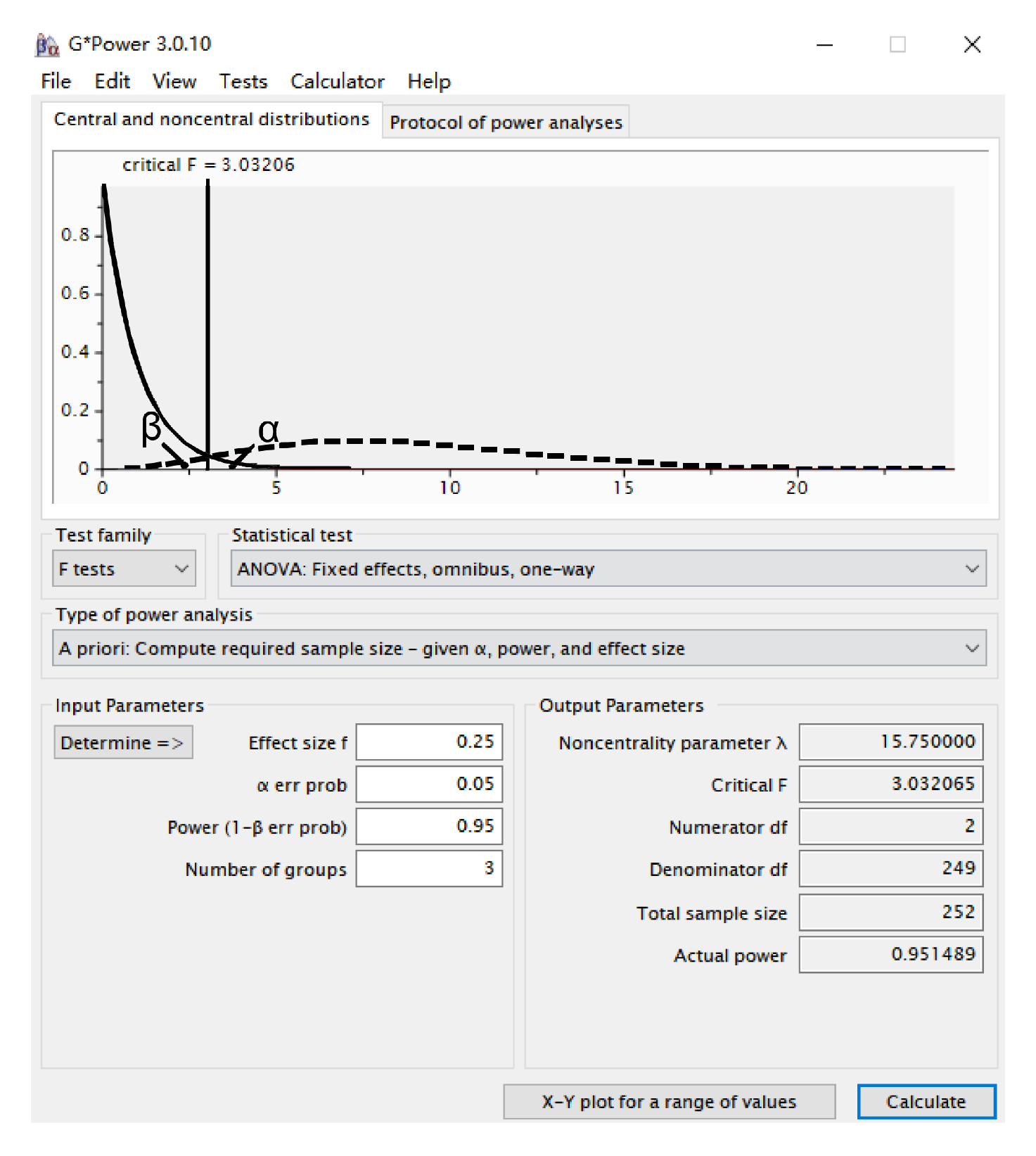

Supplement: Supplementary file 1 — Additional file 1: Supplementary Figure 1. Sample size estimation. [file 12872_2023_3628_MOESM1_ESM.docx]
