# Supplementary material for: Favorable factors for the survival of ST-segment elevation myocardial infarction patients with medium- and high-risk thrombolysis in myocardial infarction scores
Source: BMC Cardiovasc Disord. 2023 Dec 13;23:614. doi: 10.1186/s12872-023-03628-7 (PMC10720153; doi:10.1186/s12872-023-03628-7)
Supplement: Supplementary file 2 — Additional file 2: Supplementary Table 1. Comparisons of clinical data of three groups of STEMI patients. Supplementary Table 2. Comparative analysis of clinical data between surviving and dead STEMI patients with medium- and high-risk TIMI scores. [file 12872_2023_3628_MOESM2_ESM.docx]

**Supplementary Table 1** Comparisons of clinical data of three groups of STEMI patients

|  | Low-risk group (n = 195) | Medium-risk group (n = 165) | High-risk group (n = 73) | *Pa* | *Pb* |
| --- | --- | --- | --- | --- | --- |
| **Hematology** |  |  |  |  |  |
| NEUT (×10^9^/L) | 8.24 (1.87, 16.85) | 7.62 (1.70, 19.55) | 6.72 (1.40, 22.85) | 0.4499 | 0.2625 |
| MNC (×10^9^/L) | 0.63 (0.17, 2.83) | 0.64 (0.21, 2.02) | 0.58 (0.14, 2.22) | > 0.9999 | 0.7754 |
| LYMR (%) | 15.70 (3.40, 47.20) | 15.70 (2.70, 54.60) | 14.10 (1.37, 43.80) | > 0.9999 | 0.8392 |
| MNCR (%) | 6.00 (2.00, 14.50) | 6.80 (1.80, 14.60) | 6.80 (1.00, 14.00) | 0.0931 | 0.2485 |
| EOSR (%) | 0.40 (0.10, 8.20) | 0.40 (0.10, 11.90) | 0.40 (0.10, 9.00) | 0.8631 | > 0.9999 |
| MHb (pg) | 30.80 (23.80, 35.50) | 30.90 (18.60, 36.20) | 30.60 (23.00, 35.90) | > 0.9999 | > 0.9999 |
| PLT (×10^9^/L) | 209.00 (71.00, 498.00) | 201.00 (56.00, 523.00) | 208.00 (74.00, 459.00) | 0.5310 | > 0.9999 |
| MPV (fL) | 10.90 (8.70, 14.60) | 11.10 (8.90, 14.70) | 11.20 (9.30, 14.40) | 0.7943 | 0.1605 |
| PDW (%) | 13.10 (8.70, 24.30) | 13.40 (8.70, 24.00) | 13.40 (8.60, 24.10) | > 0.9999 | > 0.9999 |
| LPR (%) | 31.90 (14.00, 59.50) | 33.00 (15.90, 59.90) | 33.90 (17.90, 60.00) | > 0.9999 | 0.2371 |
| PCT (%) | 0.23 (0.07, 0.48) | 0.22 (0.08, 0.50) | 0.23 (0.08, 0.57) | 0.7443 | > 0.9999 |
| **Biochemical indicators** |  |  |  |  |  |
| ALT (U/L) | 34.00 (8.00, 706.00) | 31.00 (5.00, 1450.00) | 28.00 (5.00, 270.00) | 0.3150 | 0.0828 |
| GGT (U/L) | 33 (0, 623) | 32 (0, 539) | 30 (0, 645) | > 0.9999 | > 0.9999 |
| TnI (>25 ng/mL) | 106 (54.36%) | 84 (50.91%) | 37 (50.68%) | 0.5136 | 0.5914 |
| TP (g/L) | 64.30 (44.70, 78.00) | 64.60 (40.90, 75.90) | 63.80 (43.90, 81.00) | > 0.9999 | 0.2477 |
| HDL (mmo1/L) | 0.94 (0.39, 2.18) | 1.02 (0.41, 2.27) | 1.02 (0.46, 1.86) | 0.0708 | 0.2633 |
| Na (mmo1/L) | 139.3 (127.9, 145.8) | 139.8 (127.2, 147.7) | 139.9 (129.9, 154.2) | > 0.9999 | > 0.9999 |
| Ca (mmo1/L) | 2.17 (1.62, 2.45) | 2.15 (1.38, 2.41) | 2.15 (1.86, 2.42) | 0.1501 | 0.0555 |
| Blood sugar (mmol/L) | 6.37 (3.14, 33.71) | 6.43 (3.23, 22.74) | 6.67 (3.26, 30.44) | > 0.9999 | 0.2173 |

Note: NEUT = Neutrophil; MNC = Monocyte; LYMR = LYMR= Lymphocyte ratio; MNCR = Monocyte ratio; EOSR = Eosinophil ratio; MHb = Mean hemoglobin; PLT = Platelet; MPV = Mean platelet volume; PDW = Platelet distribution width; LPR = Platelet-large cell ratio; PCT = Platelet crit; ALT = Alanine aminotransferase; GGT = Gamma-glutamyl transpeptidase; TnI = Troponin I; TP = Total protein; HDL = High density lipoprotein; Na = sodium; Ca = calcium.

**Supplementary Table 2** Comparative analysis of clinical data between surviving and dead STEMI patients with medium- and high-risk TIMI scores

|  | Survival group (n = 215) | Death group (n=23) | *P* |
| --- | --- | --- | --- |
| **General data** |  |  |  |
| Age (Year) | 67 (28, 90) | 71 (46, 85) | 0.0643 |
| Gender  Male (n, %) | 164 (76.28%) | 14 (60.87%) | 0.1057 |
| Female (n, %) | 51 (23.72%) | 9 (39.13%) |  |
| Systolic pressure (mm Hg) | 123 (60, 199) | 119 (82, 168) | 0.3441 |
| Diastolic pressure (mm Hg) | 74 (46, 125) | 73 (52, 118) | 0.4247 |
| Time of onset (h) | 13.0 (0.5, 720.0) | 9.0 (2.0, 168.0) | 0.6248 |
| **Myocardial infarction site** |  |  | 0.8348 |
| Anterior wall (n) | 130 | 16 |  |
| Inferior wall (n) | 83 | 7 |  |
| Lateral wall (n) | 1 | 0 |  |
| Posterior wall (n) | 1 | 0 |  |
| **Hematology** |  |  |  |
| WBC (×10^9^/L) | 9.55 (3.08, 28.09) | 12.58 (4.53, 24.76) | 0.2732 |
| MNC (×10^9^/L) | 0.63 (0.15, 2.02) | 0.58 (0.14, 2.22) | 0.6951 |
| MNCR (%) | 6.80 (1.00, 14.60) | 6.20 (1.80, 14.00) | 0.3188 |
| EOSR (%) | 0.40 (0.10, 11.90) | 0.30 (0.10, 4.70) | 0.2308 |
| BASR (%) | 0.30 (0.00, 1.60) | 0.30 (0.00, 0.80) | 0.7672 |
| ERY (×10^12^/L) | 4.49 (2.19, 5.76) | 4.45 (2.30, 5.41) | 0.2998 |
| MCV (fL) | 93.40 (76.40, 107.70) | 91.70 (62.50, 105.20) | 0.1441 |
| RDW (fL) | 45.60 (35.40, 62.80) | 44.60 (37.40, 54.20) | 0.5093 |
| PLT (×10^9^/L) | 202.00 (56.00, 459.00) | 208.00 (74.00, 523.00) | 0.6029 |
| MPV (fL) | 11.10 (8.90, 14.70) | 11.10 (9.50, 14.50) | 0.4781 |
| PDW (%) | 13.40 (8.60, 23.20) | 13.50 (9.00, 24.10) | 0.6117 |
| LPR (%) | 33.50 (15.90, 59.50) | 33.10 (20.90, 60.00) | 0.4822 |
| PCT (%) | 0.22 (0.08, 0.57) | 0.24 (0.08, 0.50) | 0.2352 |
| **Biochemical indicators** |  |  |  |
| AST (U/L) | 67 (0, 1299) | 96 (0, 478) | 0.5403 |
| TnI (>25 ng/mL) | 111 (51.63%) | 10 (43.48%) | 0.4574 |
| GLB (g/L) | 25.07 ± 4.114 | 23.73 ± 4.939 | 0.1471 |
| A/G | 1.57 (0.83, 2.48) | 1.51 (1.00, 2.14) | 0.7775 |
| TG (mmo1/L) | 1.32 (0.43, 7.80) | 1.08 (0.34, 6.15) | 0.0579 |
| HDL (mmo1/L) | 1.02 (0.46, 2.24) | 1.12 (0.41, 2.27) | 0.5973 |
| K (mmo1/L) | 3.98 ± 0.45 | 4.05 ± 0.62 | 0.5106 |
| Ca (mmo1/L) | 2.15 (1.38, 2.42) | 2.12 (1.83, 2.35) | 0.4462 |
| **Coagulation indicators** |  |  |  |
| PtA (%) | 89 (37, 135) | 85 (54, 128) | 0.3384 |

Note: WBC = White blood cell; MNC = Monocyte; MNCR = Monocyte ratio; EOSR = Eosinophil ratio; BASR = Basophil ratio; ERY = Erythrocyte; MCV = Mean corpuscular volume; RDW = Red blood cell distribution width; PLT = Platelet; MPV = Mean platelet volume; PDW = Platelet distribution width; LPR = Platelet-large cell ratio; PCT = Platelet crit; AST = Aspartate aminotransferase; TnI = Troponin I; ALB = Albumin; GLB = Globulin; TG = Triglyceride; HDL = High density lipoprotein; K = Potassium; Ca = calcium; PtA = Prothrombin activity.
